# Plague and the Rat

**Published:** 1920-11-13

**Authors:** 


### Plague and the Rat.

We hear a great deal about "rat weeks," and the public has been told officially of the danger of the introduction of plague into this country through rat-infested ships. But it cannot be said that up to the present the warnings have met with an adequate response. That the danger is not to be scoffed at, however, is proved in the very able review of the problem of ship-borne plague which Dr. R. J. Reece contributes by way of an appendix to the Ministry of Health's annual report. He shows how rats have been the cause of plague on board ship, and gives the reason why the danger of infection is greater than it was before the war.

During the war a large amount of shipping was taken up for the various purposes of the belligerents. Such goods as have been carried by sea have been those most urgently required, and the total amount of tonnage of the mercantile marine has been materially reduced. The result has been that large stocks of merchandise have accumulated at certain ports abroad, waiting for an opportunity of shipment to this and to other countries. Much of this merchandise afforded excellent opportunity for the harbourage of rats, as well as ample food and facilities for nesting and breeding. Owing to the shortage of vessels and the necessity for avoiding all delays to ships, the quick voyage, and the short stay in docks, ordinary fumigation of ships for rat destruction, which prior to the war was becoming usual throughout the world, fell into abeyance. With the freeing of vessels from actual war purposes, and the resumption of trade, the accumulated stores of merchandise are being shipped from foreign ports. In all probability a greater number of rats than are usually carried with the cargoes on board ship have found their way into vessels during the latter months of the war and since the Armistice. "The result has been that plague in rats and humans on board ship has come to our notice rather frequently of late, and with it a cause of anxiety lest the disease be introduced into England." Tables are given showing lists of vessels which have arrived in this country during the last twenty-five years and on board of which cases of suspected plague among passengers and crews or among rats had occurred during the war. From these tables it may be seen that the number of such ships has varied considerably in different years, and *that the number has increased during the last few years.*

There is an interesting diagram which shows the respective percentages of the species of rats caught at four of the principal ports—London, Liverpool, Cardiff and Hull. From this diagram it will be seen that the number of brown rats caught in London and Cardiff far exceeds the number of black rats, whereas at Liverpool and Hull the black rat and Alexandrinus rat predominate. But a large number of the rats tabulated have been caught on board ship. What is important about the diagram is that it indicates the presence among ships' rats of the species of rat that is most prone to acquire, carry, and transmit the infection of plague, and reveals the desirability of destroying these rats at the point at which they can be most readily destroyed in bulk—and this is on board ship after removal of the cargo.

The actual cost of the disinfection of a large steamer—£30 or £40—is not a matter which carries much weight with a large shipping company. It is the time occupied by the process of disinfection which is considered of importance; during this time the wages of the crew have to be paid, dock dues may be incurred, and the process of loading or discharging cargo is suspended. The demurrages on a large ship may amount to as much as £1,000 a day. Nevertheless, the presence of such a disease as plague on board ship places on the shipping company concerned, and incidentally on commerce, a far heavier toll. It calls for concerted action among the maritime countries of the world to limit, as far as possible, such occurrences, not only in the interest of the shipping trade, but to prevent outbreaks of the disease in countries where plague is not present.

This principle was recognised in the Convention of 1912. It is probable that in the near future, through inter-allied action, the de-ratisation of ships at regular intervals of time will be required, and that all vessels trading in the East will be subject to a process of de-ratisation at intervals of a few months or at the end of each voyage. The situation, as it is explained in Dr. Reece's very lucid summary, suggests that after all the real method of plague prevention in this country is to be found not so much in the organisation of spectacular rat weeks ashore as in isolating ships, and, under a careful official supervision, putting them through a thorough process of de-ratisation according to the recognised methods. If this routine of extermination were universally adopted, the danger of plague would rapidly become negligible.

